# Altered GABAergic, glutamatergic and endocannabinoid signaling is accompanied by neuroinflammatory response in a zebrafish model of social withdrawal behavior

**DOI:** 10.3389/fnmol.2023.1120993

**Published:** 2023-05-22

**Authors:** Panagiotis Perdikaris, Catherine R. Dermon

**Affiliations:** Human and Animal Physiology Laboratory, Department of Biology, University of Patras, Patras, Greece

**Keywords:** NMDAR hypofunction, social deficits, anxiety, endocannabinoid system, neuroinflammation, GABAergic, glutamatergic neurotransmission

## Abstract

**Introduction:**

Deficits in social communication are in the core of clinical symptoms characterizing many neuropsychiatric disorders such as schizophrenia and autism spectrum disorder. The occurrence of anxiety-related behavior, a common co-morbid condition in individuals with impairments in social domain, suggests the presence of overlapping neurobiological mechanisms between these two pathologies. Dysregulated excitation/inhibition balance and excessive neuroinflammation, in specific neural circuits, are proposed as common etiological mechanisms implicated in both pathologies.

**Methods and Results:**

In the present study we evaluated changes in glutamatergic/GABAergic neurotransmission as well as the presence of neuroinflammation within the regions of the Social Decision-Making Network (SDMN) using a zebrafish model of NMDA receptor hypofunction, following sub-chronic MK-801 administration. MK-801-treated zebrafish are characterized by impaired social communication together with increased anxiety levels. At the molecular level, the behavioral phenotype was accompanied by increased mGluR5 and GAD67 but decreased PSD-95 protein expression levels in telencephalon and midbrain. In parallel, MK-801-treated zebrafish exhibited altered endocannabinoid signaling as indicated by the upregulation of cannabinoid receptor 1 (CB1R) in the telencephalon. Interestingly, glutamatergic dysfunction was positively correlated with social withdrawal behavior whereas defective GABAergic and endocannabinoid activity were positively associated with anxiety-like behavior. Moreover, neuronal and astrocytic IL-1β expression was increased in regions of the SDMN, supporting the role of neuroinflammatory responses in the manifestation of MK-801 behavioral phenotype. Colocalization of interleukin-1β (IL-1β) with β_2_-adrenergic receptors (β_2_-ARs) underlies the possible influence of noradrenergic neurotransmission to increased IL-1β expression in comorbidity between social deficits and elevated anxiety comorbidity.

**Discussion:**

Overall, our results indicate the contribution of altered excitatory and inhibitory synaptic transmission as well as excessive neuroinflammatory responses in the manifestation of social deficits and anxiety-like behavior of MK-801-treated fish, identifying possible novel targets for amelioration of these symptoms.

## Introduction

Impairments in social functioning are in the core of clinical symptoms characterizing many neuropsychiatric disorders, ranging from social aversion in Autism Spectrum Disorder (ASD) and schizophrenia to an unusual hyper-social phenotype observed in Williams-Syndrome (Barak and Feng, 2016). One of the proposed pathophysiological mechanisms contributing to social withdrawal is the disruption of the balance between excitation and inhibition (E/I balance), often associated with hyper-glutamatergic and/or hypo-GABAergic function at large-scale neuronal circuits (Fatemi et al., 2009; Sohal and Rubenstein, 2019). At the single-neuron level, different factors contribute to E/I imbalance including aberrant synaptic transmission and plasticity arising from altered neurotransmitter release (Takada et al., 2015), impaired expression of excitatory (Catts et al., 2016) or inhibitory postsynaptic receptors (Fatemi et al., 2009) and/or their scaffolding proteins (Coley and Gao, 2019). Such alterations cause changes in circuit excitability in key cortical and subcortical regions, contributing to social withdrawal (Zikopoulos and Barbas, 2013).

The potential involvement of N-methyl-D-aspartate receptors (NMDAR) hypofunction in the manifestation of social withdrawal phenotype has been emphasized in several studies both in rodents and teleosts, since sub-chronic (+)-MK-801 treatment, a non-competitive NMDAR antagonist, decreased social interaction in adult rats (Matsuoka et al., 2005) and zebrafish (Perdikaris and Dermon, 2022). Also, the selective knockout of NMDARs in parvalbumin interneurons resulted in decreased sociability in mice (Saunders et al., 2013), further highlighting NMDA receptors’ importance in regulating social behavior. Indeed, NMDARs located on fast-spiking GABAergic interneurons have a role of E/I imbalance in NMDA hypofunction models of social withdrawal, leading to excessive glutamate release and hyperstimulation of downstream cortical and sub-cortical networks (Homayoun and Moghaddam, 2007). Consistent with this view, it was reported increased ratio of E/I activity, in a serine racemase knockout model of NMDAR hypofunction (Jami et al., 2021). However, further studies are necessary for better understanding the pathophysiological mechanisms implicated in the synaptic dysfunction and contribute to aberrant network activity observed in neuropsychiatric disorders characterized by impairments in social functioning.

There is increasing evidence from clinical data reporting a significant link between impaired social function and anxiety, since individuals with high-functioning autism exhibited higher levels of anxiety compared to healthy control subjects (Top et al., 2016). Similarly, animal studies support the above relationship as various ASD mouse models (Peça et al., 2011; Chao et al., 2018) and MK-801-treated zebrafish (Perdikaris and Dermon, 2022) exhibit both decreased sociability as well as increased anxiety levels. Moreover, experimental manipulations that facilitate social behaviors tend to diminish anxiety levels, suggesting that certain subsets of these two pathologies may arise from common neural mechanisms. Since the disturbance of E/I balance in various brain regions can also promote anxiety-like behavior (Yuen et al., 2012; Ghosal et al., 2020; Yu et al., 2020), it is worth investigating the specific synaptic mechanisms that contribute to E/I imbalance after NMDAR hypofunction, linking social withdrawal with anxiety-like behavior.

Previous studies have demonstrated that altered inflammatory responses are associated with various neuropsychiatric disorders including anxiety and ASD (El-Ansary and Al-Ayadhi, 2014; Masi et al., 2017; Zheng et al., 2021), whereas also the levels of both pro-inflammatory and anti-inflammatory cytokines were associated with symptoms severity (Ashwood et al., 2011; Masi et al., 2017). Interestingly, interleukin 1β (IL-1β) has been shown to induce anxiety-like behavior in mice by interacting with endocannabinoid binding receptors (Rossi et al., 2012) as well as to be implicated in the etiology of ASD by contributing to the E/I imbalance (El-Ansary and Al-Ayadhi, 2014). Also, the implication of IL-1β in influencing E/I balance is further supported by the manifestation of social withdrawal phenotype following the activation of IL-1 receptor 1 (IL-1R1), located on glutamatergic neurons in hippocampus (DiSabato et al., 2021). While evidence supports that altered cytokine profile contributes to social withdrawal and anxiety-like phenotype, by regulating synaptic structure and function (Pozzi et al., 2018), further research is required to better understand the relationship between E/I imbalance and excessive inflammatory responses in the above comorbidities.

The zebrafish (*Danio rerio*) due to its well characterized repertoire of social and anxiety-like behavior and the fact that a high proportion of disease-related genes in humans possess at least one zebrafish orthologue (Howe et al., 2013), provide an attractive model to investigate the pathophysiological mechanisms that contribute to social deficits and anxiety-like behavior. Decision-making processes that are related to social behavior are regulated by the social-decision making network (SDMN), an evolutionary conserved network consisting of two interconnected circuits, the social behavior network and the mesolimbic reward system (O'Connell and Hofmann, 2011). This network is highly conserved between mammals and zebrafish sharing high structural and circuit homology thus giving the opportunity to investigate and identify specific neural circuits characterized by altered E/I balance, associating aberrant region activity with impairments in social function and anxiety-like behavior.

In the current study we used a zebrafish model displaying NMDAR hypofunction, after sub-chronic MK-801 administration in order to assess the possible influences of impaired glutamatergic and GABAergic neurotransmission in the manifestation of social withdrawal and anxiety-like behavior. Also, in order to associate impaired synaptic plasticity across the SDMN with the comorbidity of social impairment and increased anxiety levels, we analyzed the expression of IL-1β in different nodes within the SDMN to link altered cytokine profile with excitatory/inhibitory ratio. Moreover, for the first time in zebrafish, we questioned the involvement of endocannabinoid receptor 1 (CB1R), as an additional mechanism contributing to the neurochemical profile, based on the crucial role of endocannabinoid signaling in regulating both excitatory and inhibitory neurotransmission (Chevaleyre et al., 2006).

## Materials and methods

### Animals and husbandry

Wild-type, adult (3–4 months old) male zebrafish of the short-fin strain were obtained from commercial distributor and housed in 40-L tanks equipped with biological filters (one fish/L), under constant aeration and a standard 12/10 h light/dark cycle, for at least 10 days prior to testing. Water quality including temperature (26°C ± 1°C), pH value (7.0 ± 0.20) and total ammonia (≤0.01 mg/L) was continuously monitored, according to established standard of zebrafish care (Avdesh et al., 2012).

### Drug and treatment

**Figure 1:**
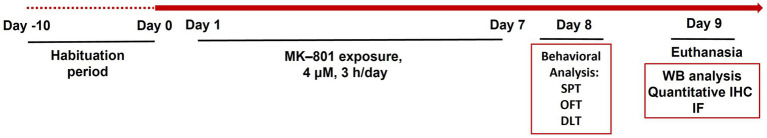
Schematic representation of the experimental timeline including the methodological approaches used to evaluate zebrafish social and anxiety-like behavior following sub-chronic MK-801 treatment. SPT, social preference test; OFT, open field test; DLT, dark–light test; WB, western blot; IHC, immunohistochemistry; IF, immunofluorescence.

(+)-MK-801 hydrogen maleate (MK-801) was purchased from Sigma Aldrich (St. Louis, Missouri, United States), dissolved in non-chlorinated water and was administered to zebrafish via water immersion for 3 h per day, for 7 consecutive days at 1,349 ng/mL (4 μΜ). Τhe dose of MK-801 was chosen based on a previous study in zebrafish (Perdikaris and Dermon, 2022). Control group was exposed to the same conditions but treated with drug-free vehicle. Immediately after the daily treatment the animals returned to their home tanks. Experimental design is shown in Figure 1.

### Behavioral assays and analysis

At the 8th day of the experimental procedure, animals were individually subjected to a battery of behavioral tests as described below. The 24 h interval period between the 7 days treatment protocol and the behavioral testing, was selected to avoid a possible acute effect of drug treatment on zebrafish behavior. All behavioral tests were conducted in the light phase of the photoperiod, between 10:00 and 15:00, and the behavioral set up was placed over a light box to improve image quality of the automated video tracking. Fish were recorded from above using a digital video camera and video recordings were analyzed using Ethovision XT9 (Noldus Inc.). The data were exported and further analyzed where needed.

### Social preference test

**Figure 2:**
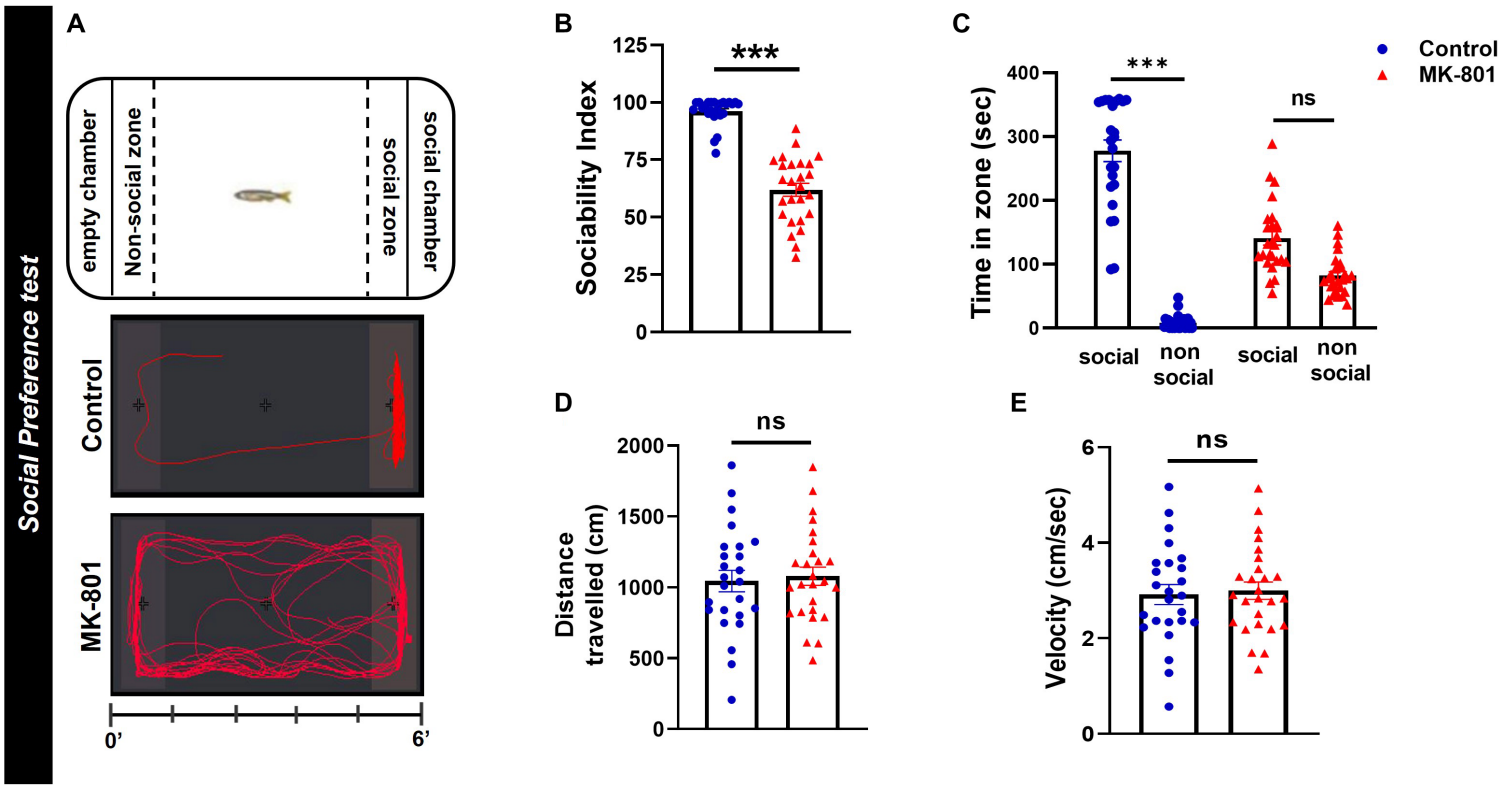
MK-801-treated zebrafish display social impairments. **(A)** Diagram of social preference test and representative behavioral trajectories of control and MK-801-treated zebrafish. **(B–E)** Social and locomotor parameters of control and MK-801-treated fish as estimated in the social preference test. **(B)** Sociability index, **(C)** time spent in social (S) and non-social (NS) zone, **(D)** distance traveled, and **(E)** mean velocity. Control: *n* = 25, MK-801: *n* = 26. Data are expressed as mean ± SEM. ^***^p ≤ 0.001, ns, non-significant, compared to control group.

The social preference test (SPT) followed the protocol described previously (Perdikaris and Dermon, 2022). Briefly, the experimental fish were placed into the center area of a glass apparatus and after an acclimatization period (150 s) were allowed to explore the tank and select between a social chamber, containing a group of four fish (1:1 sex ratio) and an empty chamber (Figure 2A), during a 6-min test. The experimental fish had visual and olfactory contact with both chambers (transparent, with 1 mm holes Plexiglas), as has also been described for rodents (Yang et al., 2011). The amount of time each test fish spent in social and non-social zone was quantified and used to calculate the sociability index [% (T_social zone_/T_social_ + T_non-social zone_)], that served as an indicator of zebrafish sociability. The social and non-social zone were defined within an area of 5 cm, immediately adjacent to the social and non-social chamber. Total distance traveled and mean velocity were also calculated.

### Open-field test

The open-field test (OFT) was used to estimate zebrafish thigmotactic behavior and locomotor activity as described elsewhere (Perdikaris and Dermon, 2022). Animals were introduced into the corner of a square arena (15 cm × 15 cm), divided into central and peripheral zone, and freely allowed to explore the tank, while their swimming activity was recorded for 6 min (Supplementary Figure 2A). The amount of time each fish spent in the peripheral zone was quantified and used to calculate the thigmotaxis index [% (T_peripheral zone_/T_arena_)], that served as an indicator of anxiety and fear-related responsiveness. Total distance traveled and mean velocity were also calculated.

### Dark–light test

**Figure 3:**
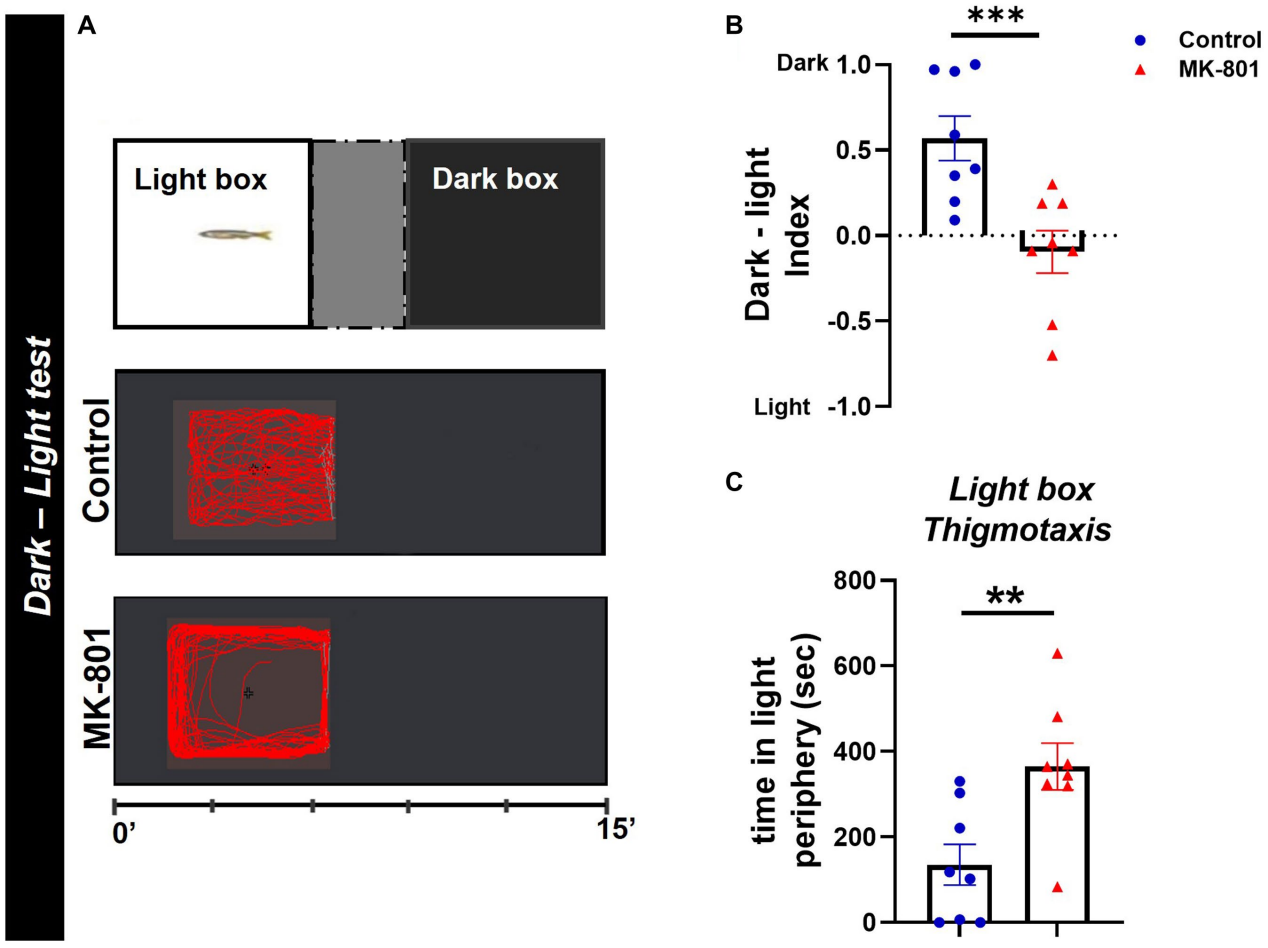
MK-801-treated zebrafish exhibit abnormal dark avoidance behavior. **(A)** Diagram of dark–light test and representative behavioral trajectories of control and MK-801-treated zebrafish. **(B,C)** Anxiety parameters of Control and MK-801-treated zebrafish as estimated in the dark–light test. **(B)** Dark–light index and **(C)** light box thigmotaxis. *n* = 8 per experimental group. Data are expressed as mean ± SEM. ^**^p ≤ 0.01, ^***^p ≤ 0.001, compared to control group.

The dark–light test (DLT), based on the rodent light/dark box (Bourin and Hascoët, 2003) was carried out here as previously described (Maximino et al., 2010a). Briefly, the experimental fish were placed into the center area of a glass apparatus and after an acclimatization period (300 s) were allowed to explore the tank for 15 min and select between two equals in size compartments, a dark and light box (Figure 3A). The walls and bottom of the boxes were covered with either dark or white opaque, non-reflective dark Plexiglas to ensure uniform background in each box. The amount of time each fish spent in dark or light box was measured and used to calculate the dark–light (DL) index (T_dark box_ − T_light box_/T_arena_). A DL index of 1 indicates 100% preference in the dark box, whereas a DL index of −1 indicates 100% preference in the light box. The proportion of time that each experimental fish displayed thigmotactic behavior in the light box (<2 cm from the walls) was also calculated and served as a risk assessment behavior (Maximino et al., 2010b).

### Western blot analysis

Animals were anesthetized, sacrificed, their brains were removed, dissected according to zebrafish brain atlas (Wullimann et al., 1996); telencephalon (without the olfactory bulbs) and midbrain (cross section 127–204), and stored at −80°C until use. Western blot was carried out under denaturing and reducing conditions as described previously (Perdikaris and Dermon, 2022). Briefly, tissues were homogenized in ice-cold RIPA lysis buffer (50 mM Tris–HCl pH 8.0, 150 mM NaCl, 1% NP-40, 0.5% sodium deoxycholate, 0.1% SDS) with 1X protease inhibitor cocktail (Roche Life Science). The homogenate was centrifuged at 5,000 rpm for 10 min at 4°C, the pellet was discarded, and the protein concentration of supernatant was determined with the BCA protein assay (BCA protein assay, Thermo Fisher Scientific).

Twenty (telencephalon) or 30 (midbrain) micrograms of total protein were separated on 9% SDS-polyacrylamide gel electrophoresis and transferred to PVDF membranes (Merck Millipore). Membranes were blocked with 5% milk in TBS-T (50 mM Trizma-Base, 150 mM NaCl, 0.05% Tween-20, pH 7.5) for 75 min, and then incubated with the appropriate primary antibodies (rabbit anti-mGluR5, mouse anti-GAD67, mouse anti-PSD95, goat anti-CB1R, mouse anti-beta-actin, Supplementary Data) diluted in 2.5% milk/TBS-T, for 16–18 h at 4°C. Membranes were washed in TBS-T, and incubated with horseradish-peroxidase-conjugated donkey anti-rabbit (1:30,000; 406,401, BioLegend), goat anti-mouse (1:15.000; A16084, Invitrogen) and donkey anti-goat (1:20.000; sc-2020, Santa Cruz) secondary antibodies at RT. Specific signals were visualized using the Immobilon Western Chemiluminescent HRP Substrate (Merck Millipore) according to the manufacturer’s instructions. Western blot films were scanned, and grayscale images were quantified by densitometric analysis using the NIH ImageJ software (National Institutes of Health). Band intensities were normalized against beta-actin that served as a loading control. Measurements were obtained in duplicate for each sample.

### Immunohistochemistry and immunofluorescence

All animals were deeply anesthetized with 0,006% tricaine methane sulfonate (MS-222, Sigma-Aldrich, E10521) and intracardially perfused with saline followed by 4% paraformaldehyde (PFA, Sigma-Aldrich, Germany) in 0.1 M phosphate buffer (PB, pH = 7.4). For the preparation of cryosections, brains were isolated, post-fixed 2 h in 4% PFA PB and cryoprotected overnight in 20% sucrose in 4% PFA at 4°C. Tissue was embedded in sectioning medium (Leica Biosystems, Buffalo Grove, United States) rapidly frozen in dry-ice-cooled isopentane (2-methylbutane; Sigma-Aldrich) at approximately −35°C and stored at −80°C. Coronal sections (20 μm thick) were cut in a Leica cryostat, collected in gelatin-coated slides, and stored at −80°C until used. After antigen retrieval with citrate buffer (10 mM Sodium citrate, 0.05% Tween 20, pH 6.0) at 80°C for 5–10 min, single or double immunohistochemistry was performed.

For single-labeling experiments, sections were incubated in 1.5% H_2_O_2_ (Sigma-Aldrich) in 0.1 M PBS for 10 min in room temperature (RT) to inhibit endogenous peroxidase activity and then washed in PBS. Non-specific protein binding sites were blocked with 5% bovine serum albumin (BSA; Sigma-Aldrich) and 1% normal horse serum (NHS; Vector Laboratories) in 0.5% Triton X-100/PBS (PBS-T) for 60 min at RT and were incubated for 16–18 h at 4°C with an antibody against IL-1 beta (IL-1β; 1:250, P420B, Invitrogen). Sections were incubated with biotinylated secondary horse anti-rabbit IgG (ready-to-use, Vector Laboratories), washed in PBS-T, incubated with Vectastain Elite ABC reagent (1:100 A and 1:100 B; Vector Laboratories) in 0.5% PBS-T for 1 h in the dark at RT and washed with PBS, followed by peroxidase-catalyzed polymerization of 3,3-diaminobenzidine (DAB; Vector Laboratories) to visualize the immunoreaction. The sections were then dehydrated with ethanol, cleared with xylene and cover slipped with Entellan (Merck, Darmstadt, Germany).

For double-labeling experiments, after the antigen retrieval step, sections were blocked, and incubated with a mixture of primary antibodies diluted in 1% BSA and 0.2% NHS in 0.5% PBS-T, for 16–18 or 40 h at 4°C. After PBS washes, slides were incubated for 2.5 h at room temperature with AlexaFluor-488 donkey anti rabbit IgG and AlexaFluor-555 donkey anti mouse IgG (1:500, Invitrogen) in 1% BSA in 0.5% PBS-T, washed again five times for 10 min each in PBS and cover-slipped with an aqueous fluorescent mounting medium (H-1700; Vector Laboratories).

### Microscopic observation and quantification of IL-1β positive cells

Brain areas of interest were determined using the zebrafish neuroanatomical atlas (Wullimann et al., 1996). Images of single and double-labeling experiments were acquired using a colored digital camera CFW-1308C (Scion Corp., United States), attached to a Nikon Eclipse E800 optical and fluorescent microscope (Nikon, Tokyo, Japan). Each image consisted of a stack of optically sliced images, generated by NIH ImageJ software, and further used for identification of double-labeled cells and/or quantification of IL-1β^+^ cells.

For quantification of IL-1β^+^, three coronal brain sections were analyzed manually per brain area, blindly to the experimental condition, using the ImageJ software. More specifically, one (Dm, PM) or two (SRF) squares (0.01 mm^2^) within each nucleus of interest per brain hemisphere, were used to quantify the number of IL-1β^+^ cells.

### Data analysis

Normal distribution of data was assessed using Shapiro–Wilk test and analyzed using the unpaired Student’s *t*-test in case of homogeneity, whereas in case of non-normal distribution by the non-parametric Mann–Whitney *U*-test for significance between “control” and “MK-801” groups. To analyze paired data (weights), the paired-samples t-test or the non-parametric Wilcoxon signed-ranked test was used. Statistical analysis was performed the statistical program IBM SPSS. Two-way analysis of variance (ANOVA), followed by Bonferroni post-test (independent variables: treatment and zone) was applied for the comparison of the time spent in the social or non-social zones by control and experimental groups. The Spearman correlation coefficient determined the strength of monotonic relationship between parameters of zebrafish behavioral and neurochemical phenotype, with effect sizes above 0.60 or below −0.60, representing strong correlations. Data are presented as mean ± standard error of the mean (SEM). Statistical significance was considered at *p* value < 0.05.

## Results

### Reduced body weight in MK-801-treated zebrafish

We compared body weights in each group (control, MK-801) at day 0 and day 9 of the experimental timeline (Figure 1). MK-801 administration caused a significant decrease in zebrafish body weight (Z = −3.701, *p* ≤ 0.001, Wilcoxon signed-ranked test). No alteration was observed in control group (Z = −1.846, *p* = 0.065, Wilcoxon signed-ranked test; Supplementary Figure 1A). No difference in body length was observed between control and MK-801-treated group (U = 228.5, *p* = 0.427, Mann–Whitney *U*-test, two tailed; Supplementary Figure 1B).

### Impaired social preference in MK-801-treated zebrafish

MK-801 treated zebrafish displayed impaired social preference compared to control group, as measured by the decreased sociability index (U = 30, *p* ≤ 0.001, Mann–Whitney *U*-test, two tailed; Figure 2B). More specifically, control zebrafish spent significantly increased time in the social than in the non-social zone whereas MK-801 treated fish showed no significant preference (treatment X zone: *F*_1,98_ = 130.979, *p* < 0.001, effect of treatment: *F*_1,98_ = 13.056, *p* = 0.001, effect of zone: *F*_1,98_ = 234.193, *p* < 0.001, Social_control_ vs. Non-Social_control_: *p* < 0.001, Social_MK-801_ vs. Non-Social_MK-801_: *p* = 0.0533, two-way ANOVA; Figure 2C). When their locomotor activities were compared, MK-801-treated and control fish traveled similar distance [*t*(49) = −0.348, *p* = 0.730, unpaired *t*-test; Figure 2D] and had similar mean velocities [*t*(49) = −0.294, *p* = 0.770, unpaired *t*-test; Figure 2E] in the social preference test. These results suggest that MK-801 fish exhibit a social withdrawal phenotype which could not be attributed to a locomotor defect, as they display similar locomotor activity with the control group.

### MK-801-treated zebrafish displayed thigmotactic behavior

We next evaluated whether MK-801 fish exhibit abnormal behavior in response to a stress-inducing stimulus, using the OFT. In comparison to the control group, MK-801-treated zebrafish displayed thigmotactic behavior as indicated by their increased thigmotaxis index (U = 25, *p* ≤ 0.001, Mann–Whitney *U*-test, two tailed; Supplementary Figure 2B), increased latency to enter the central zone (U = 75, *p* ≤ 0.001, Mann–Whitney *U*-test, two tailed; Supplementary Figure 2C), and decreased total number of entries to the center (U = 45.5, *p* ≤ 0.001, Mann–Whitney *U*-test, two tailed; Supplementary Figure 2D), suggesting the presence of increased anxiety. No significant alteration was observed in the locomotor parameters between control and MK-801 fish (mean velocity: U = 195, *p* = 0.904; distance traveled: U = 194, *p* = 0.883, Mann–Whitney *U*-test, two tailed; Supplementary Figures 2E,F).

### MK-801-treated zebrafish displayed abnormal avoidance behavior in the DLT

We next performed the DLT as another measure of anxiety-like behavior. This assay revealed that MK-801 fish displayed a preference for the light box, as indicated by the decreased dark–light index [*t*(14) = 3.689, *p* = 0.002, unpaired *t*-test; Figure 3B], compared to control group. Moreover, MK-801-treated fish exhibited a characteristic thigmotactic behavior in the light box, as they spend more time in the periphery of the light box, compared to control [*t*(14) = −3.166, *p* = 0.007, unpaired *t*-test; Figure 3C]. Regarding their locomotor activity in the light box, no significant difference was observed in mean velocity between both groups [*t*(14) = −0.669, *p* = 0.515, unpaired *t*-test] whereas MK-801-treated fish traveled increased distance compared to control [*t*(14) = −3.767, *p* = 0.002, unpaired *t*-test; data not shown]. These data suggest that MK-801-treated zebrafish display a characteristic dark avoidance behavior, which may serve as a readout for increased anxiety levels, as also has been indicated in previous studies (Champagne et al., 2010).

### Altered expression levels of proteins that are implicated in E/I balance, in telencephalon and midbrain of MK-801-treated zebrafish

**Figure 4:**
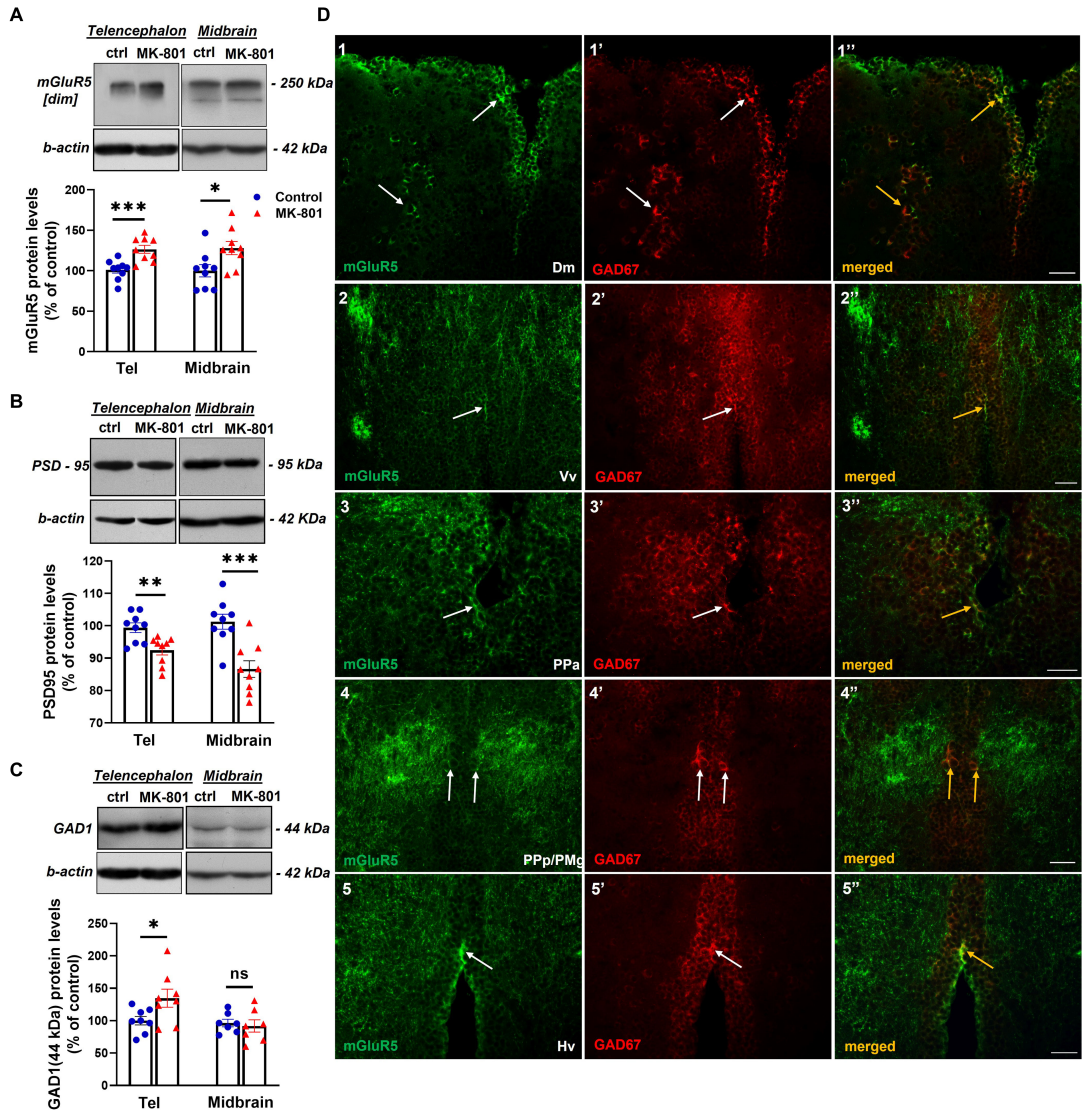
MK-801-treated zebrafish are characterized by altered expression levels of proteins involved in glutamatergic and GABAergic neurotransmission **(A–C)** Representative blots and quantification of the relative expression levels of **(A)** mGluR5, **(B)** PSD-95 and **(C)** GAD1. *n* = 6–7 (GAD1) or *n* = 9 (mGlur5, PSD-95) per experimental group. Data are expressed as mean ± SEM. ^*^p ≤ 0.05, ^**^p ≤ 0.01, ^***^p ≤ 0.001, ns, non-significant, compared to control group. **(D)** Immunofluorescent microphotographs of selected transverse sections showing colocalization of mGluR5 with GAD1 immunoreactive cells within different areas of the SDMN. **(D1–1’’)** medial zone of the dorsal telencephalic area (Dm), **(D2–2’’)**- ventral nucleus of the ventral telencephalic area (Vv), **(D3–3’’)** parvocellular preoptic nucleus, anterior part (PPa), **(D4–4’’)** parvocellular preoptic nucleus, posterior part/gigantocellular part of magnocellular preoptic nucleus (PPp/PMg), **(D5–5’’)** ventral zone of periventricular hypothalamus (Hv). Arrows indicate examples of colocalization. Microphotographic images are representative of both control **(D1–1”,D3–3”,D4–4”,D5–5”)** and MK-801-treated **(D2–2’’)** fish. Scale bar: 25 μm.

The disruption of E/I balance is one of the proposed mechanisms implicated in social withdrawal (Gao and Penzes, 2015), thus we questioned whether MK-801-treated fish exhibited altered expression levels of proteins that regulate excitatory and inhibitory neurotransmission. We first analyzed the expression levels of mGluR5, a key modulator of glutamatergic neurotransmission and synaptic plasticity (Piers et al., 2012). mGluR5 was detected as a single band at approximately 250 kDa, corresponding to its dimeric functional form (Lum et al., 2016) and considered as specific in previous studies using the same antibody (Ayala et al., 2012). MK-801-treated fish displayed increased total mGluR5 protein expression levels in telencephalon [*t*(16) = −3.994, *p* = 0.001, unpaired *t*-test] and midbrain [*t*(16) = −2.537, *p* = 0.022, unpaired *t*-test; Figure 4A], compared to control group. Next, we analyzed the protein levels of PSD-95 (postsynaptic density protein 95), a major postsynaptic scaffold protein in the PSD of glutamatergic synapses, that is involved in synaptic plasticity and neuronal excitability (Kim and Sheng, 2004; Prange et al., 2004; Keith and El-Husseini, 2008) and its deficiency is associated with several neuropsychiatric disorders (Matosin et al., 2016; Coley and Gao, 2018). Interestingly, our results indicate that MK-801-treated fish displayed decreased protein expression levels of PSD-95 in telencephalon [*t*(16) = 3.329, *p* = 0.004, unpaired *t*-test] and midbrain [*t*(16) = 4.241, *p* ≤ 0.001, unpaired *t*-test; Figure 4B] compared to control. In addition to the alterations observed in glutamatergic markers, we analyzed the protein expression levels of GAD1, the key enzyme that catalyzes the conversion of L-glutamic acid into the neurotransmitter GABA (Martin and Barke, 1998). More specifically, our antibody recognized a more intense band at 44 kDa, corresponding to an enzymatically active isoform of GAD1 as characterized both in mouse and zebrafish brain (Hariharan, 2013; Trifonov et al., 2014). Our results indicate that the protein expression levels of GAD1(44 kDa) were significantly increased in telencephalon of MK-801-treated fish [*t*(14) = −2.242, *p* = 0.042, unpaired *t*-test], whereas no significant alterations were observed in midbrain [*t*(12) = 0.400, *p* = 0.696, unpaired *t*-test; Figure 4C].

**Figure 5:**
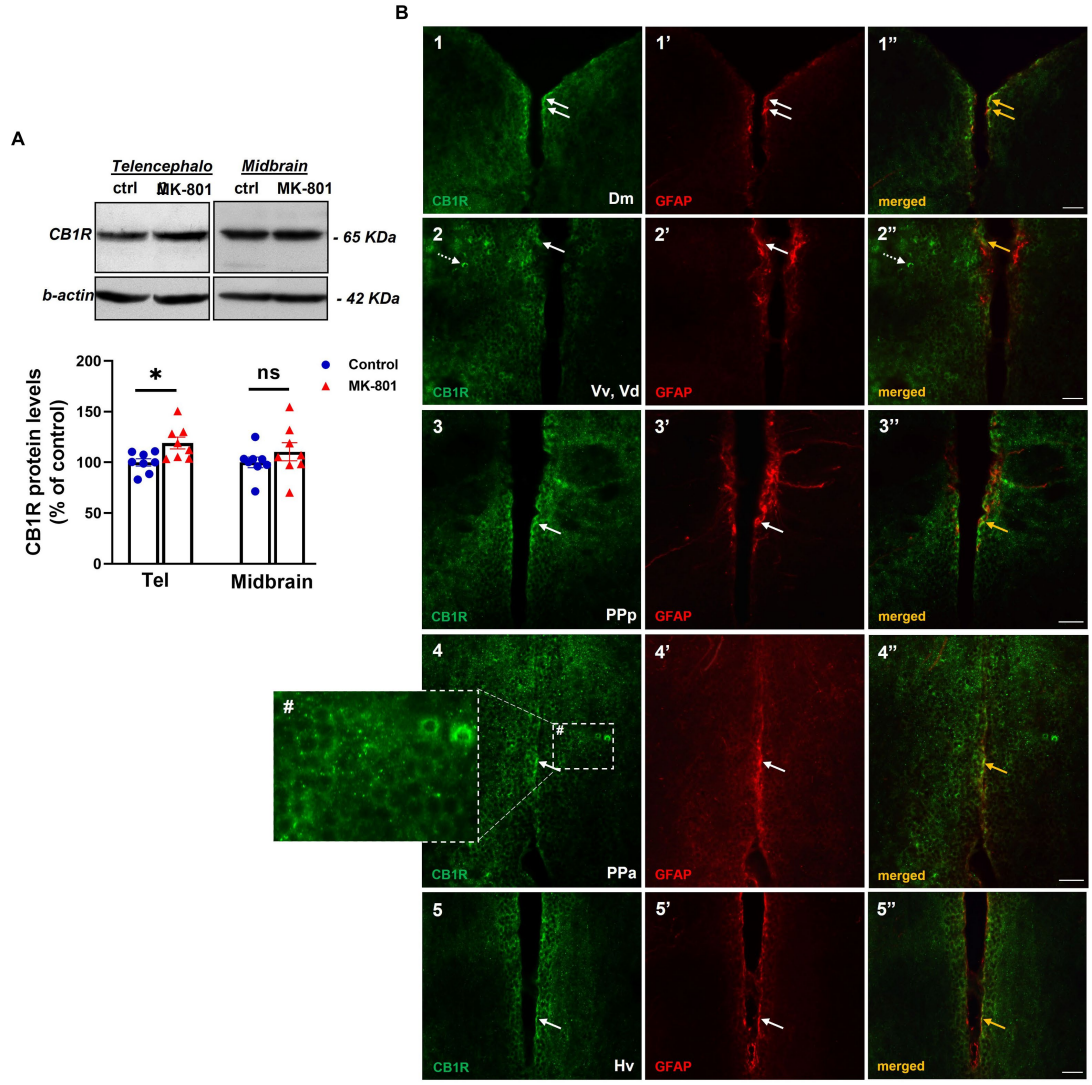
Altered endocannabinoid signaling in telencephalon of MK-801-treated zebrafish. **(A)** Representative blots and quantification of the relative expression levels of CB1R in telencephalon and midbrain. *n* = 8 per experimental group. Data are expressed as mean ± SEM. ^*^p ≤ 0.05, ns, non-significant, compared to control group. **(B1–5’’)** Immunofluorescent microphotographs of selected transverse sections showing colocalization of CB1R with GFAP immunoreactive cells in the periventricular zone within different nodes of the SDMN. **(B1–1’’)** medial zone of the dorsal telencephalic area (Dm), **(B2–2’’)** ventral nucleus of the ventral telencephalic area/dorsal nucleus of the ventral telencephalic area (Vv/Vd), **(B3–3’’)** parvocellular preoptic nucleus, anterior part (PPa), **(B4–4’’)** parvocellular preoptic nucleus, posterior part (PPp), **(B5–5’’)** ventral zone of periventricular hypothalamus (Hv). Arrows indicate examples of colocalization, dashed arrows point to single positive cells. Within brain parenchyma, CB1R-immunostaining is intensively present in punctate structures around or in close proximity with cell somata **(B4#)**. Microphotographic images are representative of MK-801-treated fish. Scale bar: 25 μm.

Given the post-synaptic localization of mGluR5 (Cartmell and Schoepp, 2000), we next analyzed whether glutamatergic activity may modulate GABAergic neurotransmission in specific forebrain areas belonging to the social decision—making network (SDMN; O'Connell and Hofmann, 2011; Figure 4D). Double immunofluorescent experiments demonstrated the localization of mGluR5 in GAD1 positive cells in the medial zone of the dorsal telencephalic area (Dm; Figure 4D1–1’’), the ventral nucleus of the ventral telencephalic area (Vv; Figure 4D2–2’’), the anterior part of parvocellular preoptic nucleus (PPa, Figure 4D3–3’’), the magnocellular preoptic nucleus (PM; data not shown), the gigantocellular part of magnocellular preoptic nucleus (PMg; Figure 4D4–4’’) and the ventral zone of periventricular hypothalamus (Hv; Figure 4D–5”). Interestingly, the high level of colocalization between mGluR5 and GAD1, in Dm, Vv and PPa was observed both in the periventricular areas as well as in the brain parenchyma. These results suggest that mGluR5 signaling may influence GABAergic neurotransmission within the SDMN, by controlling the excitability of GAD1^+^ cells.

### MK-801-treated zebrafish are characterized by altered CB1R protein expression levels

The protein expression levels of CB1R served as an indirect indication of endocannabinoid system activity. Specifically, we observed increased protein expression levels of CB1R in telencephalon of MK-801-treated fish [*t*(14) = −2.802, *p* = 0.014, unpaired *t*-test], whereas no significant difference was observed in midbrain [*t*(14) = −1.018, *p* = 0.326, unpaired *t*-test; Figure 5A].

CB1R immunoreactivity was intensively present in punctate structures or in cell bodies and to a lesser extent in fibers, in forebrain areas belonging to the SDMN (Figure 5B1–5’’). Interestingly, double labeling experiments showed that CB1R/GFAP co-expression was mainly localized in the periventricular area of Dm (Figure 5B1–1’’), ventral and dorsal nucleus of the ventral telencephalic area (Vv, Vd; Figure 5B2–2’’), posterior part of parvocellular preoptic nucleus (PPp, Figure 5B3–3’’), PPa (Figure 5B4–4’’) and Hv (Figure 5B5–5’’). As expected, CB1R immunoreactive puncta in the parenchyma were not colocalized with GFAP, as they may represent excitatory and inhibitory boutons (Figure 5B4#). These data raise the possibility that altered CB1R signaling may regulate neuronal excitability and synaptic transmission directly, by inhibiting neurotransmitter release in the presynaptic terminal or indirectly through the Ca^2+^-release of gliotransmitters and subsequent modulation of synaptic activity in heteroneuronal synapses, based on the “tripartite synapse” concept (Perea et al., 2009).

### Abnormal IL-1β expression and neuronal/glial phenotype of IL-1β^+^ cells within the SDMN of MK-801-treated zebrafish

**Figure 6:**
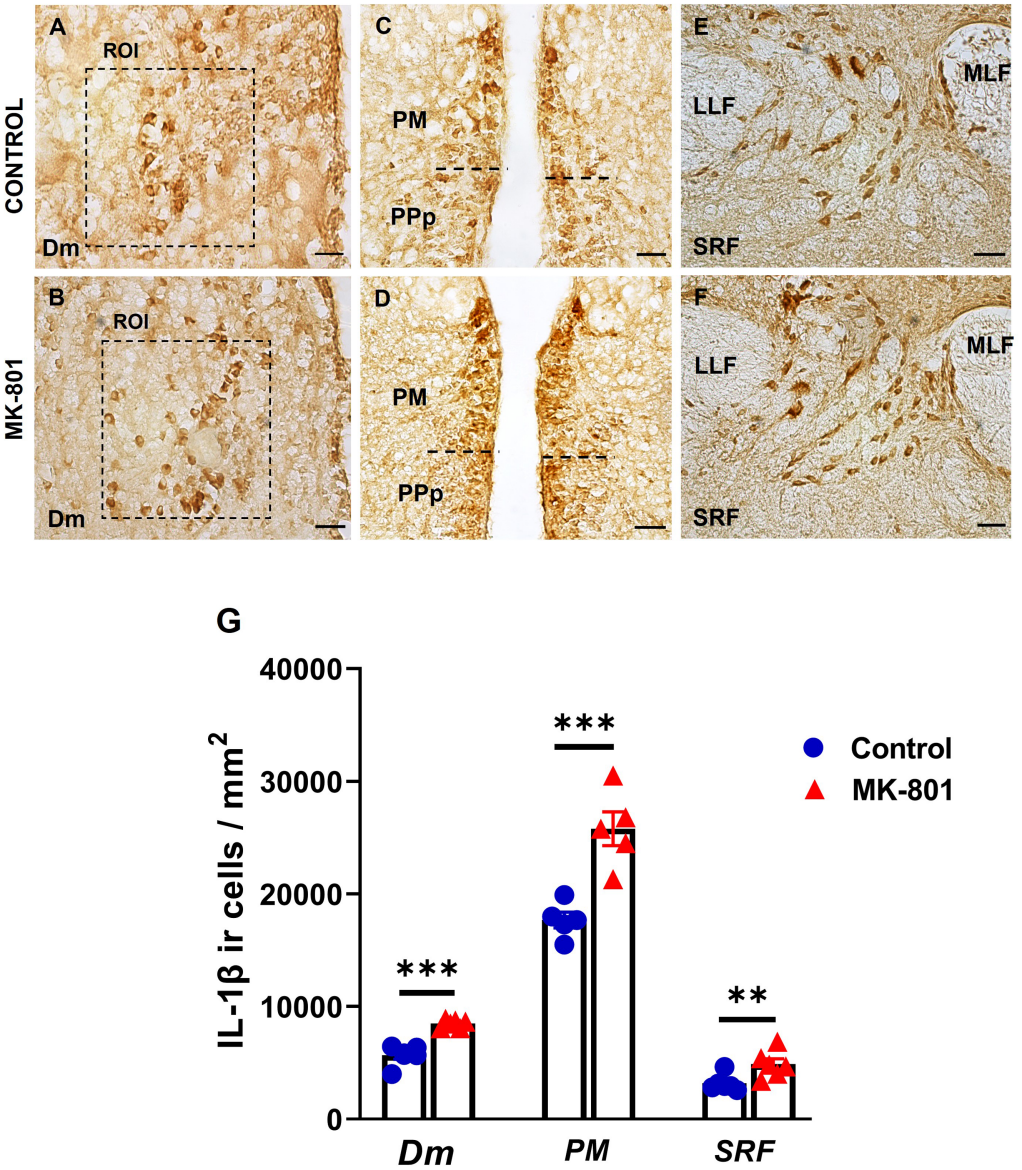
Excessive IL-1β expression within the SDMN and SRF, in MK-801 treated zebrafish displaying social withdrawal and anxiety-like behavior. **(A–F)** Representative staining for IL-1β in **(A,B)** medial zone of the dorsal telencephalic area (Dm), **(C,D)** magnocellular preoptic nucleus and **(E,F)** superior reticular formation (SRF) of control and MK-801 treated fish. Scale bar: 25 μm. **(G)** The density of IL-1β positive cells were significantly increased in MK-801 treated zebrafish, in Dm, PM and SRF, compared to control group. Bars represent the number (mean ± SEM) of IL-1β positive cells. *n* = 5 per experimental group. ^**^p ≤ 0.01, ^***^p ≤ 0.001, compared to control group.

The density of IL-1β positive cells was quantified in selected brain areas belonging to the SDMN; medial zone of the dorsal telencephalic area (Dm), magnocellular preoptic nucleus (PM) and in midbrain structures (Figures 6A-F) known to regulate arousal states: superior reticular formation (SRF; Faraguna et al., 2019). Our results revealed that MK-801-treated zebrafish displayed significantly increased density of IL-1β positive cells within the region of interest (ROI) in Dm [*t*(8) = −5.946, *p* ≤ 0.001], PM [*t*(8) = −4.886, *p* = 0.001], and SRF [*t*(8) = −4.406, *p* = 0.008] compared to control group (Figure 6G), suggesting that excessive IL-1β expression within the SDMN, may be associated with social withdrawal behavior and increased anxiety levels.

**Figure 7:**
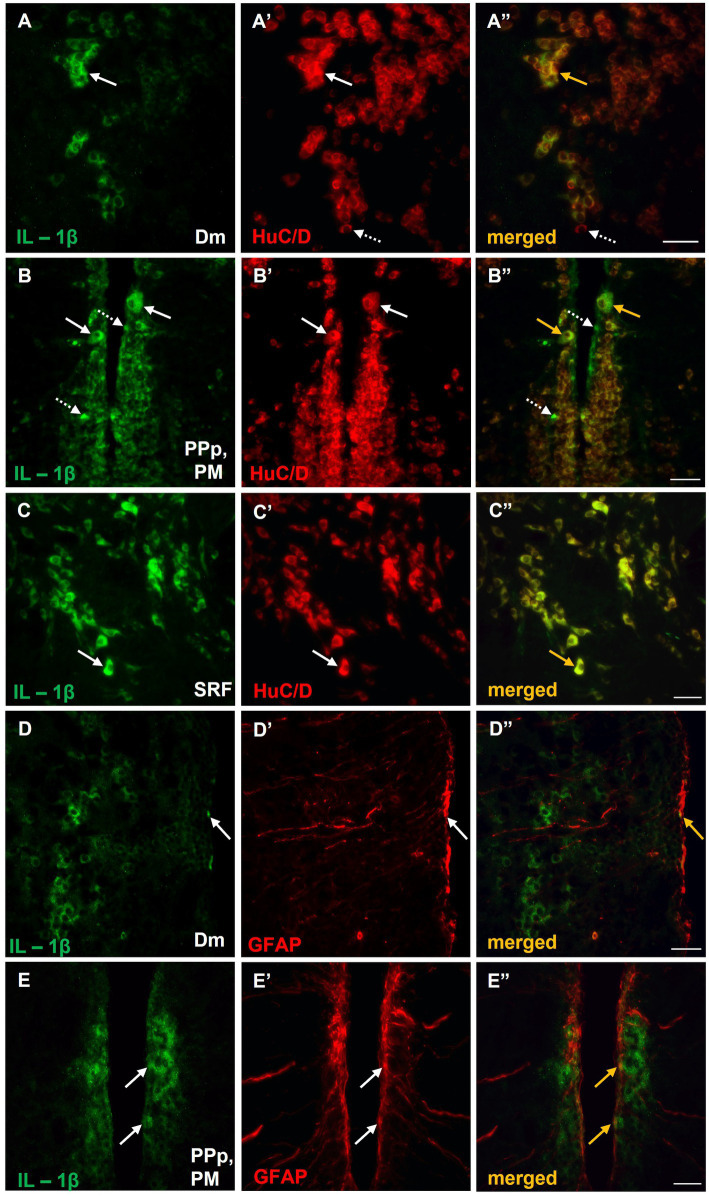
Expression of IL-1β in neurons and/or astrocytes within areas of the SDMN, in MK-801-treated zebrafish. **(A–E’’)** Immunofluorescent microphotographs of selected transverse sections showing colocalization of IL-1β with HuC/D **(A–C”)**, or GFAP **(D–E”)** in medial zone of the dorsal telencephalic area (Dm) **(A–A”,D–D”)**, parvocellular preoptic nucleus, posterior part/magnocellular preoptic nucleus (PPp, PM) **(B–B”,E–E”)** and superior reticular formation (SRF) **(C)**. Arrows indicate examples of colocalization, dashed arrows point to single positive cells. Microphotographic images are representative of MK-801-treated fish, *n* = 5. Scale bar: 25 μm. ROI, region of interest.

The identity of IL-1β^+^ cells in the aforementioned regions of the SDMN was determined by double immunofluorescence experiments with pan–neuronal (HuC/D) and glial markers (GFAP). The vast majority of IL-1β positive cells in Dm (Figure 7A–A’’), PM (Figure 7B–B’’), and SRF (Figure 7C–C’’) were HuC/D^+^ neurons. However, IL-1β was colocalized with GFAP almost exclusively in the periventricular zone of Dm (Figure 7D–D’’) and PM (Figure 7E–E’’). Specifically, within the region of interest of Dm parenchyma, a few, small GFAP^+^ cells that also expressed IL-1β were found (Figure 7D–D’’). No colocalization between IL-1β and GFAP was observed in SRF (data not shown).

**Figure 8:**
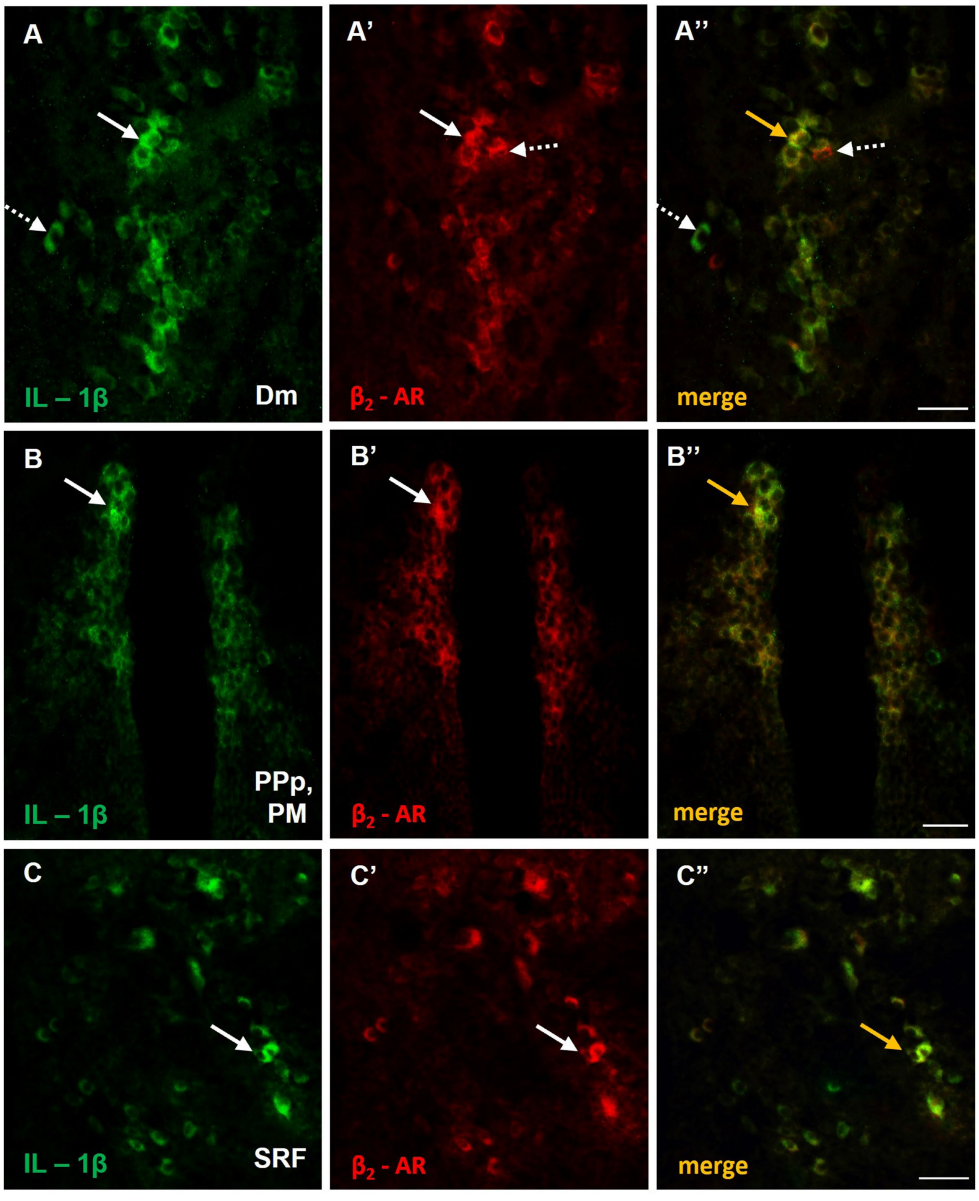
Possible noradrenergic regulation of IL-1β expression through β_2_-adrenoceptors (β_2_-ARs) activation within areas of the SDMN. **(A–C’’)** Immunofluorescent microphotographs of selected transverse sections showing colocalization of IL-1β with β_2_-ARs in **(A–A’’)** medial zone of the dorsal telencephalic area (Dm), **(B–B’’)** parvocellular preoptic nucleus, posterior part/magnocellular preoptic nucleus (PPp, PM), and **(C–C’’)** superior reticular formation (SRF). Arrows indicate examples of colocalization, dashed arrows point to single positive cells. Microphotographic images are representative of MK-801-treated fish, *n* = 5. Scale bar: 25 μm.

To further investigate the possible involvement of microglia in the abnormal immune response, observed in MK-801-treated zebrafish, we performed double immunofluorescence staining with OX-42 (CD11b), a general marker expressed in activated microglia (Jeong et al., 2013). No expression levels of OX-42 were detected in the forebrain and midbrain of control zebrafish (data not shown) whereas in MK-801-treated fish, a few small IL-1β positive cells in Dm (Supplementary Figure 3A) and SRF (Supplementary Figure 3B) also expressed OX-42. No OX-42 immunoreactive microglia cells were observed in PM of MK-801-treated fish (data not shown). Interestingly, immunofluorescence analysis demonstrated that IL-1β^+^ cells expressed also β_2_-adrenoceptors (β_2_-ARs) in Dm (Figures 8A–A’’), PPp/PM (Figure 8B–B’’) and SRF (Figure 8C–C’’). Together, these results suggest that neurons and astrocytes, but not microglial cells, are primarily involved in increased expression pattern of IL-1β, within SDMN in MK-801-treated group, and highlight a possible noradrenergic influence on the expression of proinflammatory responses.

### Social withdrawal and anxiety-like behavior are related to distinct mechanisms

**Figure 9:**
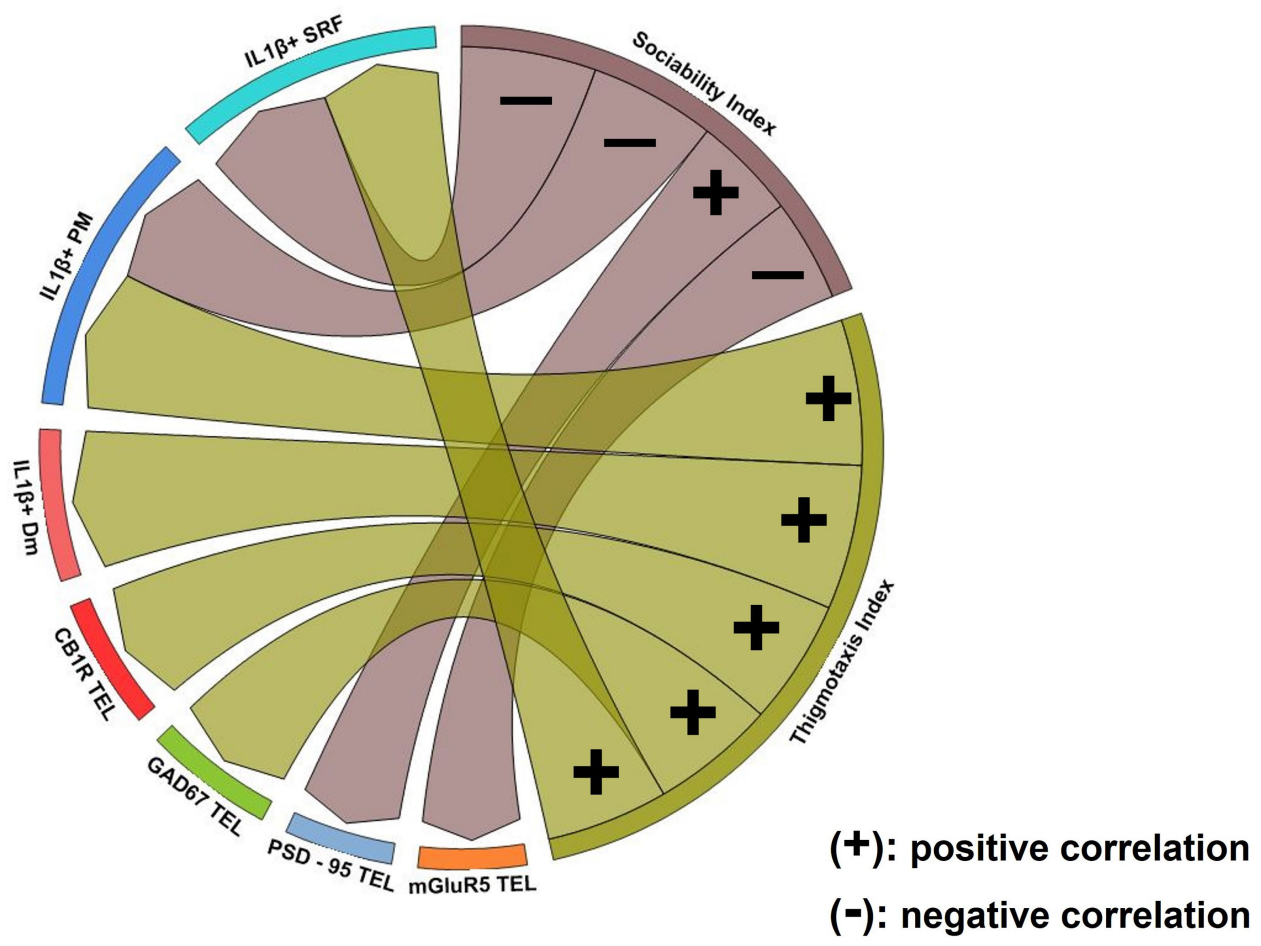
Chord diagram obtained from Spearman’s correlation analysis showing the relationship between social withdrawal and anxiety-like behavior, excitation/inhibition imbalance and excessive IL-1β expression. Links indicate strong positive (+) and negative (−) significant correlations, with “*r*” values above 0.6 or below −0.6, respectively. Notice that glutamatergic dysfunction was associated with social withdrawal behavior whereas GABAergic and endocannabinoid system dysfunction was associated with increased anxiety. Excessive IL-1β expression in Dm was associated with anxiety-like behavior while in PM and SRF was linked both to social withdrawal and increased anxiety. *p* ≤ 0.05.

Spearman pair-wise correlations determined the possible relationship between the behavioral phenotype in MK-801-treated fish and their telencephalic glutamatergic and GABAergic activity (Figure 9; Supplementary Table 1). Interestingly, the sociability index was negatively correlated with telencephalic mGluR5 expression and positively correlated with PSD-95 expression levels. No correlation was observed between thigmotactic behavior and elements of glutamatergic neurotransmission. Importantly, thigmotactic behavior was positively correlated with GAD1 and CB1R protein expression levels in the telencephalon of MK-801 group, indicating that glutamatergic and GABAergic neurotransmission contribute differentially to aspects of the behavioral profile observed in MK-801-treated fish.

The excessive IL-1β expression within areas of the SDMN were related with the asocial and anxiety-like behavior in MK-801-treated fish (Figure 9; Supplementary Table 1). Our results indicate that the density of IL-1β^+^ cells in Dm was positively correlated with thigmotaxis index, whereas no correlation was observed with sociability index. In PM, the number of IL-1β^+^ cells were negatively correlated with sociability index and positively correlated with thigmotactic behavior. Similarly, the density of IL-1β^+^ cells in SRF was negatively correlated with sociability index and positively correlated with thigmotactic behavior. These results suggest that excessive IL-1β expression in Dm is possibly associated with anxiety-like behavior whereas the altered IL-1β expression profile in PM and SRF may be implicated in the manifestation of both social withdrawal and anxiety-like state.

## Discussion

The present study advanced previous evidence (Perdikaris and Dermon, 2022) on MK-801-treated zebrafish characterized by impaired social communication together with increased anxiety levels, a common comorbid psychiatric symptom in individuals diagnosed with several neuropsychiatric disorders, such as ASD and schizophrenia (Top et al., 2016; Hall, 2017). Additionally, at the molecular level these behavioral defects were accompanied by altered expression levels of different proteins associated with glutamatergic and GABAergic neurotransmission, thus implying the possible presence of E/I imbalance at synaptic or circuit level in MK-801-treated fish. Such imbalance is suggested to represent a common pathophysiological mechanism implicated in the etiology of social deficits (Gao and Penzes, 2015). Importantly, the dysregulated GABAergic and glutamatergic neurotransmission was accompanied by increased telencephalic expression of CB1R protein levels and increased density of IL-1β^+^ cells in specific forebrain and midbrain nuclei, also highlighting the presence of endocannabinoid and immune system dysregulation. Τhis is of particular significance since several studies, both in humans as well as in rodents have reported alterations in endocannabinoid signaling as well as immune dysfunction in various conditions characterized by social impairment (Careaga et al., 2017; Zou et al., 2021; Robinson-Agramonte et al., 2022), thus further validating this zebrafish pharmacological model as a useful model for studying the mechanisms involved in social dysfunction.

MK-801-treated zebrafish displayed robust social deficits as indicated by the decreased sociability index. More specifically, MK-801-treated fish did not show any characteristic preference for the social zone compared to the non-social compartment, supporting previous findings of impaired social interaction in rodents and zebrafish after acute and sub-chronic MK-801 administration (Rung et al., 2005; Gururajan et al., 2012; Zimmermann et al., 2016; Perdikaris and Dermon, 2022). Although deficits in social communication commonly co-occur with increased anxiety, a possible common neural mechanism remains largely unclear, with studies reporting that the “basolateral amygdala-ventral hippocampus” circuitry is involved in regulating both behaviors (Felix-Ortiz and Tye, 2014). Similarly, MK-801-treated zebrafish displayed increased anxiety levels as indicated by their increased thigmotaxis index in the OFT, a well-validated index of fear and anxiety in zebrafish (Maximino et al., 2010b). Moreover, the increased latency of MK-801-treated fish to enter the central compartment as well as the decreased center entries serve as further indicators of their centrophobic behavior. Previous studies are consistent with this view, since NMDA receptor hypofunction after MK-801 administration, caused heightened anxiety state in male rats, as indicated by increased thigmotactic behavior (Feinstein and Kritzer, 2013) or the presence of hypoactivity and decreased exploratory behavior (Latysheva and Rayevsky, 2003) in the OFT.

In order to have a better understanding on zebrafish anxiety state, we analyzed their behavior in the DLT, a well-established paradigm for estimating anxiety-levels based on a conflict between their preference for protected areas and their innate motivation to explore new environments (Maximino et al., 2010a). Our results indicated that MK-801-treated fish spent increased time in the light compartment compared to control group, as indicated by the dark–light index. Although, most of the studies have interpreted this behavior as anxiolytic (Lau et al., 2011; Duarte et al., 2019; Fontana et al., 2022) other findings characterize dark-avoidance behavior as an anxiety-like response (Champagne et al., 2010). The latter may appear paradoxical, but one must consider that zebrafish are diurnal species and unlike nocturnal rodents, moving to a lit area may be preferable in order to avoid predators (Ziv et al., 2013). It should also be noted that our control group displayed a characteristic dark preference/light avoidance behavior similar to other studies reporting the innate preference of zebrafish for dark vs. light environments (Maximino et al., 2010a). In an attempt to carefully elucidate this, we propose that the observed light preference of MK-801-treated zebrafish, accompanied by thigmotactic behavior, may indicate an adaptive risk assessment behavior, an anxiety-related response. Risk assessment behavior is based on individual’s inability to determine if there is a threatening situation or environment and subsequent HPA axis activation (Blanchard et al., 1990; Blanchard, 2018). In agreement, a previous study suggested that the exploration of white compartment in the DLT is primarily driven by fear/anxiety and not novelty, due to the absence of intrasession habituation (Maximino et al., 2010a). Also, the anxiolytic properties of isoflavones on zebrafish were demonstrated in another study, after decreasing their thigmotactic behavior in the light compartment (de Melo et al., 2020). Increased number of risk-assessment behaviors were also observed in BTBR mice, an animal model of ASD also characterized by impaired sociability and high anxiety (Chao et al., 2018). In line with the above, adult zebrafish that were subjected to social isolation during their early-life stages displayed a characteristic dark avoidance behavior that could not be interpreted as an anxiolytic response, suggesting the inability to develop stress-associated arousal state (Varga et al., 2020). Our perspective that MK-801-treated zebrafish are characterized by a heightened anxiety-state, is further reinforced by their diminished weight gain, a response that is reported in rodents and zebrafish after prolonged stress exposure (Riaz et al., 2015; Fulcher et al., 2017). In support, MK-801-treated zebrafish were previously shown to exhibit aspects of anxiety-like behavior in the novel tank test (Perdikaris and Dermon, 2022). Although new behavioral endpoints and may provide additional information about the behavioral phenotype of MK-801 treated fish, our data support the presence of a robust social withdrawal and anxiogenic profile, characterizing many neuropsychiatric disorders.

E/I imbalance is considered an important pathophysiological mechanism contributing to social withdrawal behavior (Rubenstein and Merzenich, 2003; Gao and Penzes, 2015). Increased evidence highlights the presence of glutamatergic dysfunction in the development of many neuropsychiatric disorders, characterized by social impairments and increased anxiety (Nasir et al., 2020; Nisar et al., 2022). In support, the imbalance of Ε/Ι in synaptic or circuit levels has been reported in several NMDA receptor hypofunction models (Flores-Barrera et al., 2020; Jami et al., 2021). In the present study, western blot results demonstrated alterations in brain neurochemistry regarding the function and stabilization of excitatory and inhibitory synapses, possibly contributing to alterations in E/I balance. Specifically, the protein expression levels of mGluR5 were significantly increased in the telencephalon and midbrain of MK-801-treated fish whereas PSD-95 protein expression levels were significantly decreased in both regions. In agreement, the pivotal role of mGluR5 signaling in social withdrawn behavior has been previously introduced in zebrafish (Perdikaris and Dermon, 2022). Studies using ASD-associated Shank3 knockout mice, that exhibit social deficits, demonstrated disrupted ability of hippocampal neurons to express mGluR1 and mGluR5, and impaired mGluR-dependent long-term depression (LTD; Lee et al., 2019), as well as increased mGluR5 expression levels in hippocampus, thalamus, and amygdala (Cai et al., 2019). On the other hand, there is increasing evidence that the mGluR5 agonists or the mGluR5 positive allosteric modulator, enhanced social interaction by normalizing NMDA receptor function in Shank2 mutant mice (Won et al., 2012), thus implying that deviation of mGluR5 activity in either direction leads to social impairments. Our results further validate the increased mGluR5 expression in telencephalon and midbrain of MK-801-treated fish, possibly indicating mGluR5 hyperactivity and that administration of mGluR5 antagonists may have beneficial effects in restoring social function.

Evidence of compromised glutamatergic neurotransmission is further supported by the downregulation of PSD-95 in the telencephalon and midbrain of MK-801-treated zebrafish. Prior studies demonstrated that PSD-95 is involved in the molecular organization of the PSD (Chen et al., 2011) and synapse stabilization by regulating the recruitment and trafficking of NMDARs and AMPARs in the postsynaptic membrane (Chen et al., 2015). Indeed, disruption of PSD-95 expression is reported in various neuropathologies that are characterized by social impairment (Matosin et al., 2016; Coley and Gao, 2018) as well as in anxiety-related responses (Feyder et al., 2010; Wang et al., 2020). More specifically, a significant decrease in PSD-95 mRNA and protein expression levels was reported in prefrontal cortex and CA1 hippocampal region in postmortem brain samples from schizophrenic patients (Ohnuma et al., 2000; Matosin et al., 2016). We suggest that the decreased expression of PSD-95 in telencephalon and midbrain of MΚ-801-treated fish observed here, may be related to an overall reduction of NMDAR and/or AMPAR in the postsynaptic membranes, leading to aberrant PSD synaptic activity.

Increasing evidence supports that dysfunction of inhibitory neurotransmission is implicated in the pathophysiology of several neuropsychiatric disorders characterized by social impairment and anxiety (Fogaça and Duman, 2019; Zhao et al., 2021). To assess whether MK-801-treated zebrafish were characterized by GABAergic system dysregulation, we analyzed the expression of GAD1 (44 kDa), an enzymatically active isoform of GAD1 that catalyzes the conversion of L-glutamic acid into the neurotransmitter GABA (Trifonov et al., 2014). Although previous studies reported decreased mRNA and protein expression levels of GAD2 and GAD1 in cerebellum and parietal cortex in autistic individuals (Fatemi et al., 2002; Yip et al., 2007) or reduced GABA_A_ receptor binding in hippocampus and cingulate cortex (Blatt et al., 2001; Oblak et al., 2009), in the present study the protein expression levels of GAD1 (44 kDa) were significantly increased in the telencephalon of MK-801-treated fish. Our results, despite the fact that they do not appear to be in the same direction with previous studies reporting suppressed GABAergic inhibition in ASD or schizophrenia (Banerjee et al., 2013; Xu and Wong, 2018), highlight a more complex picture since enhanced glutamatergic activation and GABAergic inhibition in different microcircuits in specific telencephalic regions of MK-801-treated fish may contribute to social impairments and increased anxiety. In agreement with our results, previous studies reported increased GABAergic activity in striatum of rodent ASD and/or fragile X syndrome model (Centonze et al., 2008; Horder et al., 2018), a finding that is indicative of the heterogeneity of mechanisms that contribute to social deficits. It is possible that the increased expression of GAD1 (44 kDa) in the telencephalon of MK-801-treated fish serves as a compensatory mechanism to increased mGluR5 signaling. Indeed, a high GAD1 and mGluR5 co-expression pattern was observed in various SDMN nodes, such as Dm, Vv, PPa, PPp/PM and Hv, supporting the hypothesis that glutamate may influence the typical GABAergic firing pattern, via mGluR5 activation. In support, GAD1 expression is known to be modulated by neuronal activity (Patz et al., 2003; Lau and Murthy, 2012), therefore the observed upregulation of GAD1 protein expression, may represent a homeostatic mechanism to counteract the increased excitability of projection neurons and thus control network activity. While the present study cannot elucidate whether MK-801 administration does indeed contribute to E/I imbalance, we hypothesize that NMDAR hypoactivity in GABAergic interneurons and/or glutamatergic projection neurons (Bygrave et al., 2019) may lead to a compensatory increase in mGluR5 and GAD1 expression and possibly to changes in circuit excitability. Although, previous studies emphasize the role of NMDAR hypofunction specifically on PV^+^ interneurons in reduced sociability (Saunders et al., 2013; Cao et al., 2022), the contribution of mGluR5 and GAD1 co-expressing cells within nodes of SDMN may underlie the comorbidity between social deficits and anxiety-like behaviors, observed in the present study.

In addition, CB1Rs expression in zebrafish SDMN and their possible involvement in the manifestation of social withdrawal and anxiety-like behavior, was addressed in the present study, taking into account that the endocannabinoid system regulates excitatory and inhibitory synaptic transmission by mediating short- and long-term of plasticity at glutamatergic and GABAergic synapses (Chevaleyre et al., 2006). Indeed, evidence is provided on the presence of disrupted endocannabinoid signaling as indicated by the increased protein expression levels of CB1R in the telencephalon of MK-801-treated zebrafish, in agreement to previous findings reporting molecular alterations in components of endocannabinoid signaling in several psychiatric conditions characterized by social impairment (Zou et al., 2021) as well as in anxiety disorders (Maldonado et al., 2022). The increased protein expression levels of CB1R in the telencephalon of MK-801-treated fish may serve as homeostatic mechanism to decreased endocannabinoid signaling, as previously described in ASD children and VPΑ-treated rats (Aran et al., 2019; Zou et al., 2021). Interestingly, a previous study reported that Fmr1 knockout mice, that are characterized by excessive mGluR5 signaling (Mody et al., 2021), displayed diminished 2-arachidonoyl-sn-glycerol (2AG)-dependent LTD at excitatory synapses of mouse forebrain (Jung et al., 2012). Moreover, the endocannabinoid system controls the activity of NMDARs (Sánchez-Blázquez et al., 2014) and as a consequence altered endocannabinoid signaling may cause excessive NMDAR hypofunction. Importantly, in the present study, the localization of CB1Rs in astroglial cells within forebrain regions belonging to the SDMN raises the possibility that may influence astrocyte activity, thus regulating the Ca^2+^-dependent gliotransmitter release and in turn modulating neuronal excitability, as an active part of the “tripartite synapse” concept (Perea et al., 2009). A recent study by Wang et al., supports the above assumptions since impaired Ca^2+^ signaling in astrocytes resulted in diminished release of ATP and contributed to ASD-like behavior (Wang et al., 2021). Considering the above, the identification of specific neural circuits characterized by aberrant E/I balance as well as altered endocannabinoid signaling may provide important synaptic endpoints of impaired neurotransmission in order to link social deficits with increased anxiety levels.

Importantly, the presence of excessive IL-1β expression in MK-801-treated fish, as indicated by the increased number of IL-1β^+^ cells colocalized with NeuN or GFAP, within the SDMN is of particular significance. In contrast to the neuronal or astroglial phenotype of IL-1β^+^ cells, only a small proportion expressed OX-42 in Dm and SRF, suggesting a minor contribution of activated microglia in the enhanced IL-1β expression within the SDMN. The contribution of neutrophils and monocytes also cannot be excluded since they also express OX-42 (Jeong et al., 2013). In agreement, excessive neuroinflammation is an element of neuropathology across many neuropsychiatric disorders characterized by social impairment and increased anxiety (El-Ansary and Al-Ayadhi, 2014; Müller et al., 2015). More specifically, IL-1β has been reported to be elevated in plasma of children and adults with ASD (Ashwood et al., 2011; Masi et al., 2017) as well as in the brain of schizophrenic patients (Söderlund et al., 2009) whereas enhanced IL-1β expression has also been implicated in anxiety-like responses in rodents (Rossi et al., 2012) and zebrafish (Song et al., 2018). Our results support the view of a dysregulated proinflammatory state in MK-801 treated fish, that is mediated by neurons and astrocytes and are consistent with a previous study reporting increased IL-1β immunoreactivity in glia cells and neurons, in structures of limbic system under stress conditions (Badowska-Szalewska et al., 2009). Similarly, increased IL-1β expression, specifically in neuronal populations of hippocampal CA1 region, striatum and paraventricular nucleus were reported in mice subjected to repeated immobilization stress (Kwon et al., 2008). Several studies have begun to investigate the non-immunological role of IL-1β, indicating that IL-1β regulates neuronal plasticity by impairing LTP in several regions of hippocampus and regulating neurotransmitter release (Merali et al., 1997; Hoshino et al., 2017) or affecting synapse structure and function, by decreasing PSD-95 expression and frequency of miniature excitatory postsynaptic currents (mEPSCs), through a MeCP2 dependent mechanism (Tomasoni et al., 2017). There is also increased evidence that excessive IL-1β disrupts the E/I balance, both by enhancing glutamatergic transmission (Mandolesi et al., 2013) or by delaying excitatory to inhibitory switch of GABA in the offspring of a maternal immune activation mouse model (Corradini et al., 2018). Therefore it is important to further link neural circuits displaying aberrant activity, with excessive IL-1β expression within SDMN regions, in MK-801-treated fish. Toward that direction, Dm a putative homologous to the mammalian basolateral amygdala (BLA) and PM, a part of the preoptic area, are key nodes in the SDMN (O'Connell and Hofmann, 2011), regulate both social behavior (Bickart et al., 2014; Nunes et al., 2021) as well as fear and anxiety-related responses (Lal et al., 2018; Chuang et al., 2021), so it is logical to considered as targets of great importance. The enhanced expression of IL-1β may alter synaptic plasticity in these brain regions, contributing to social impairments and anxiety-like behavior, observed in MK-801-treated fish. Similarly, it was recently shown that excessive inflammatory response induced anxiety and depressive-like behavior by increasing the excitability of BLA glutamatergic neurons (Zheng et al., 2021). Consistent with this interpretation, excessive IL-1β production in Dm was positively correlated with thigmotactic behavior, providing further evidence about the implication of this region in regulating anxiety responses. Moreover, heightened inflammatory responses in SRF, a region implicated in regulation of arousal states (Faraguna et al., 2019), may contribute to the behavioral profile of MK-801-treated fish, since abnormally elevated arousal is associated with stress and anxiety (Pfaff et al., 2007). Indeed, IL-1β expression in SRF was positively correlated with social withdrawal and anxiety-like behavior, supporting the notion that SRF may be part of a common network regulating both behaviors.

While we did not extensively determine the phenotype of IL-1β + cells, our data regarding the localization of β_2_-adrenoceptors (β_2_-ARs) in the IL-1β expressing neurons or astrocytes, raise the possibility of a direct noradrenergic regulation of IL-1β production. Such noradrenergic influence has been reported via modulation of the cAMP cell levels (Bourne et al., 1974; Vizi, 1998). The induction of IL-1β by β_2_-AR signaling has been previously observed in a mouse model of social defeat stress, where the increased expression of IL-1β mRNA in microglia cells was prevented by a β-ΑR antagonist (Wohleb et al., 2011). The above interpretation does not exclude the possibility that additional neurotransmission systems, such as glutamate, are implicated in excessive proinflammatory responses, since previous studies reported increased IL-1β production following prolonged neuronal activity (Del Rey et al., 2016). However, the present data combined with previous evidence on increased β_2_-AR protein expression levels in the forebrain and midbrain of MK-801-treated zebrafish (Perdikaris and Dermon, 2022) support a possible contribution of noradrenergic neurotransmission in the excessive IL-1β expression. Nevertheless, further studies are needed in order to investigate the above assumption and associate it with the comorbidity between social deficits and increased anxiety levels.

## Conclusion

In the present study the possible presence of dysregulated of excitatory and inhibitory neurotransmission is highlighted in forebrain and midbrain of a zebrafish pharmacological model, displaying robust social impairment and increased anxiety-like behavior. Specifically, glutamatergic dysfunction was positively associated with social withdrawal behavior whereas impaired GABAergic signaling was linked with anxiety-like behavior. In addition, altered endocannabinoid signaling accompanied the manifestation of these behaviors, suggesting the possible regulatory role of CB1Rs in astrocytes.

Moreover, the presence of excessive IL-1β expression in neurons and astroglia, within key nodes of the SDMN, as well as the colocalization with β_2_-ARs, is suggested to serve as an additional pathophysiological mechanism contributing to social withdrawal and anxiety-like behavior. Evidence provided here using zebrafish, confirm and expand previous rodents’ studies providing an additional insight into the putative contribution of altered E/I balance and exaggerated proinflammatory response in the pathophysiology of social deficits and anxiety-like behavior.

## Data availability statement

The raw data supporting the conclusions of this article will be made available by the authors, without undue reservation.

## Ethics statement

All experimental procedures were approved by the Ethics Committee of University of Patras and were in accordance with the European Communities council directive (86/609/EEC) for the care and use of laboratory animals. All efforts were made to minimize animal suffering and to reduce the number of animals used.

## Author contributions

PP and CD conceived and designed experiments and wrote and reviewed the manuscript. PP performed the experiments and analyzed the data. All authors contributed to the article and approved the submitted version.

## Funding

Part of the work was supported by the Hellenic Foundation for Research and Innovation (HFRI) scholarship to PP. The publication fees of this manuscript have been financed by the Research Council of the University of Patras.

## Conflict of interest

The authors declare that the research was conducted in the absence of any commercial or financial relationships that could be construed as a potential conflict of interest.

## Publisher’s note

All claims expressed in this article are solely those of the authors and do not necessarily represent those of their affiliated organizations, or those of the publisher, the editors and the reviewers. Any product that may be evaluated in this article, or claim that may be made by its manufacturer, is not guaranteed or endorsed by the publisher.
