# Supplementary material for: Altered GABAergic, glutamatergic and endocannabinoid signaling is accompanied by neuroinflammatory response in a zebrafish model of social withdrawal behavior
Source: Front Mol Neurosci. 2023 May 22;16:1120993. doi: 10.3389/fnmol.2023.1120993 (PMC10239971; doi:10.3389/fnmol.2023.1120993)
Supplement: Supplementary file 1 [file Data_Sheet_1.docx]

***Supplementary Material***

***Supplementary Data***

**The following primary antibodies were used**:

**WB**: rabbit anti-mGluR5 (1:1000 for telencephalon, 1:1500 for midbrain; AB5675, Merck-Millipore), mouse anti-GAD67 (1:700; MAB5406; Merck-Millipore), mouse anti-PSD95 (1:500, sc-32290, Santa Cruz), goat anti-CB1R (1:1000, NB100-2427, Novus Biologicals) and mouse beta-actin (1:10.000, sc-47778, Santa Cruz)

**IHC, IF**: The following primary antibodies were used: polyclonal rabbit anti-mGluR5 (1:500, AB5675, Merck-Millipore), monoclonal mouse anti-GAD67 (1:250; MAB5406; Merck-Millipore), polyclonal rabbit anti-CB1R (1:200; 209550, Merck-Millipore), polyclonal rabbit anti-IL 1β (1:250, P420B, Invitrogen), monoclonal mouse anti-GFAP (1:1000; clone G-A-5, G3893, Sigma-Aldrich), mouse monoclonal anti- HuC/D (1:100; clone 16A11, A21271, Invitrogen). Antibodies specificity was validated with negative controls that included: (i) omission of primary or secondary antibody or (ii) application of secondary antisera mismatched for species. No labeling was observed in any case.

***Supplementary Figures and Tables***


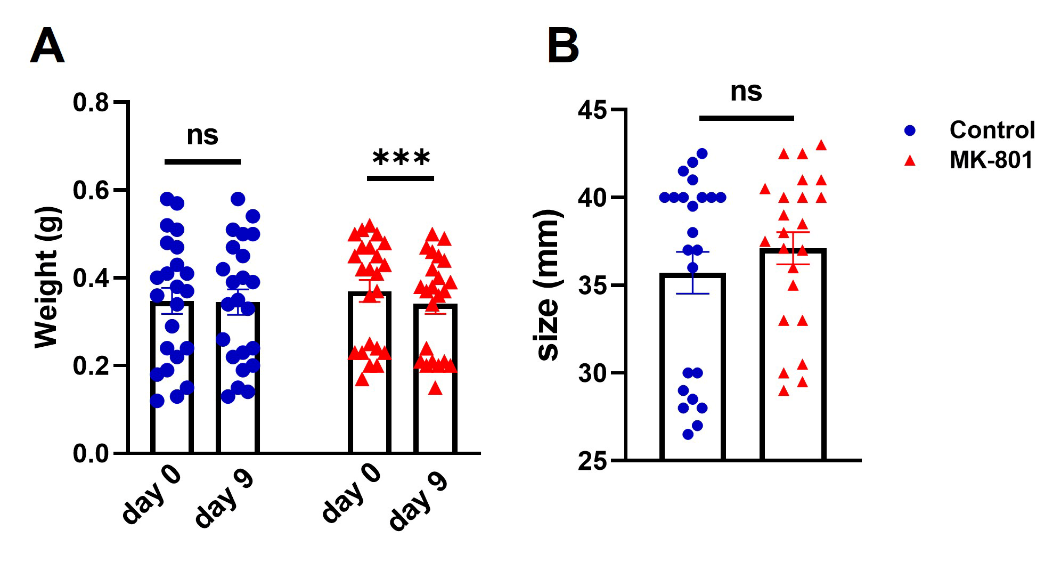


**Supplementary Figure 1:** (**A**) Decrement in zebrafish body weight was observed, only after MK-801-treatment. (**B**) No alteration in body length was observed between control and MK-801-treated zebrafish, (n=23 per experimental group)


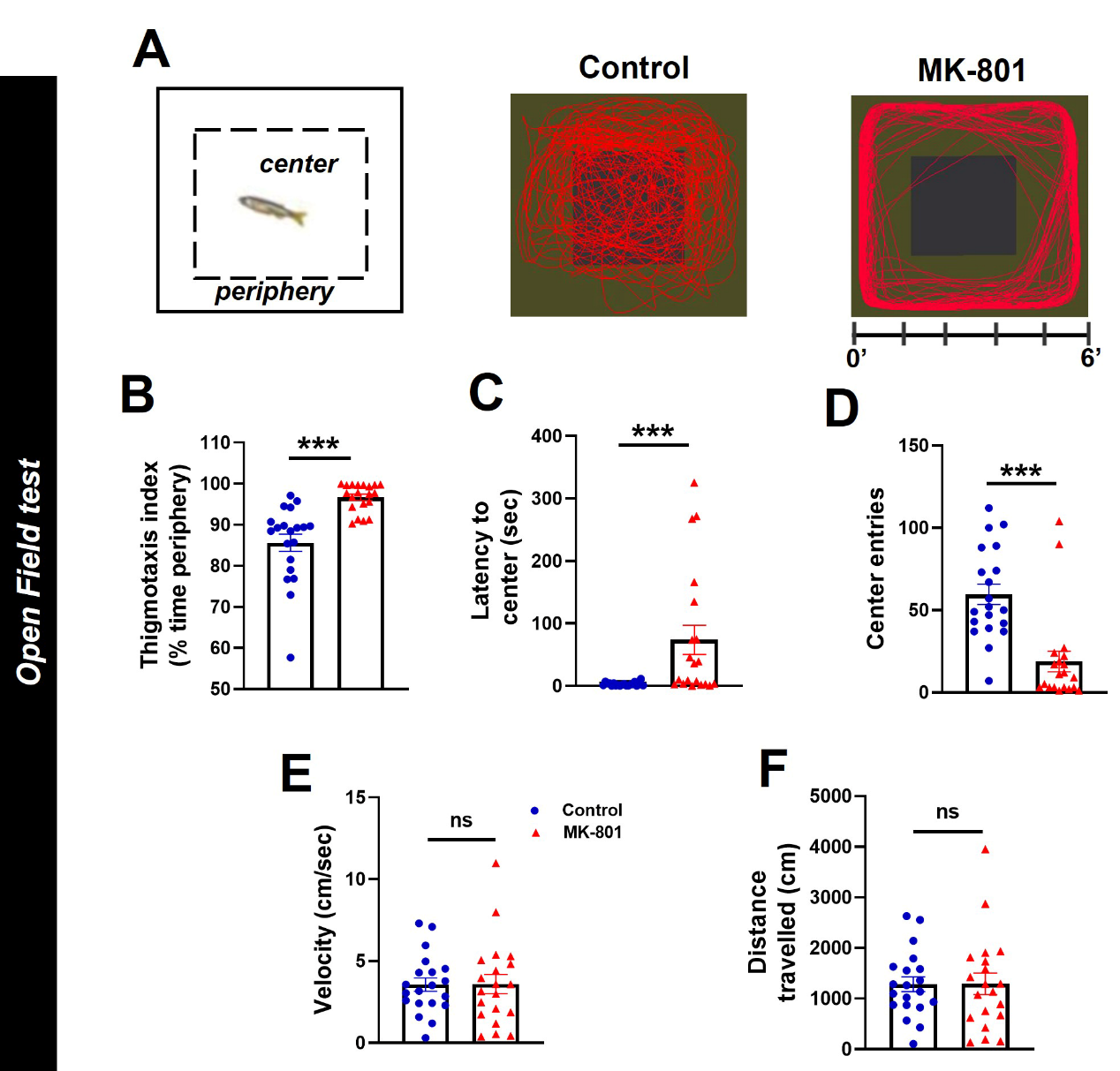


**Supplementary Figure 2:** MK-801-treated zebrafish display anxiety-like behavior. (**A**) Diagram of the open field test and representative behavioral trajectories of control and MK-801-treated zebrafish. (**B-F**) Anxiety and locomotor parameters of control and MK-801-treated fish as estimated in the open field test. (B) thigmotaxis index, (C) latency to center, (D) entries to center, (E) mean velocity and (F) distance travelled. (**G**) Diagram of dark-light test and representative behavioral trajectories of control and MK-801-treated zebrafish. n=20 per experimental group. Data are expressed as mean±SEM. ***p≤0.001, ns: non-significant, compared to control group.


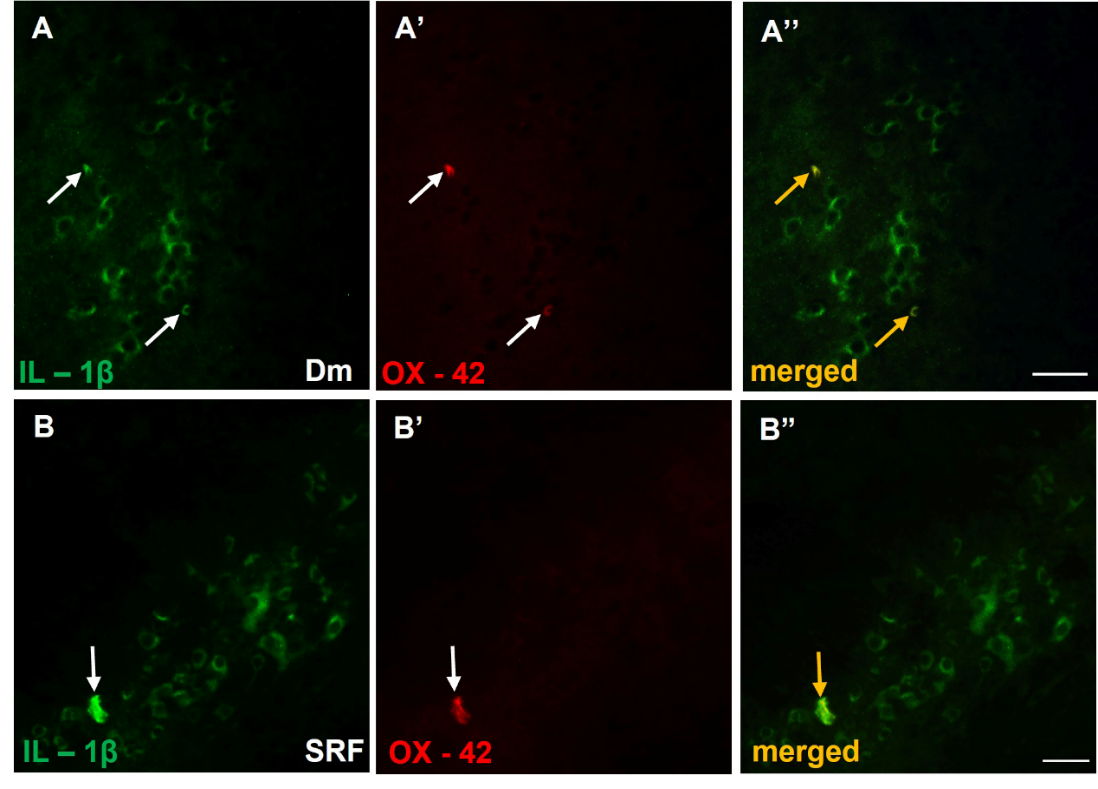


**Supplementary Figure 3**. Low contribution of activated microglia in the excessive IL – 1β expression within areas of the SDMN, in MK – 801 treated zebrafish. (**A, B**) Immunofluorescent microphotographs of selected transverse sections showing colocalization of IL - 1β with OX - 42 in medial zone of the dorsal telencephalic area (Dm) (A) and superior reticular formation (SRF) (B). Arrows indicate examples of colocalization. Microphotographic images are representative of MK – 801 – treated zebrafish n=5. Scale bar: 25 μm.

**Supplementary Table 1:** Values of Spearman’s Rank Correlation Coefficient, r_s_

|  | ***Sociability Index*** | ***Thigmotaxis Index*** |
| --- | --- | --- |
| ***mGluR5 TEL*** | **-0.639 **,**  **p =0.003** | **0.343,**  **p=0,150** |
| ***PSD – 95 TEL*** | **0.644**, p=0.004** | **-0.219,**  **p=0,459** |
| ***GAD67 TEL*** | **-0.291,**  **p=0.274** | **0.771*** p≤0.001** |
| ***CB1R TEL*** | **-0.394, p=0.131** | **0.776**,**  **p=0.003** |
| ***IL-1β^+^ cells Dm*** | **-0.638, p=0,064** | **0.898**,**  **p=0.002** |
| ***IL-1β^+^ cells PM*** | **-0.783*, p=0.013** | **0,903***, p≤0.001** |
| ***IL-1β^+^ cells SRF*** | **-0.836**, p=0,005** | **0.758*,**  **p=0.011** |
